# Identification of Two Rare Variants in Iranian Families With Familial Sudden Cardiac Death

**DOI:** 10.1155/ijog/5965922

**Published:** 2026-01-07

**Authors:** Mahsa Tahmasebivand, Sepideh Mehvari, Fatemeh Ghodratpour, Hamidreza Khoram Khorshid, Reza Malekzadeh, Reza Najafipour, Yasser Riazalhosseini, Mark Lathrop, Hossein Najmabadi, Kimia Kahrizi

**Affiliations:** ^1^ Genetics Research Center, University of Social Welfare and Rehabilitation Sciences, Tehran, Iran, uswr.ac.ir; ^2^ Digestive Disease Research Institute, Shariati Hospital Tehran University of Medical Sciences, Tehran, Iran, tums.ac.ir; ^3^ Victor Phillip Dahdaleh Institute of Genomic Medicine, McGill University, Montreal, Canada, mcgill.ca

**Keywords:** channelopathies, rare variant, *RYR2*, *SCN5A*, sudden cardiac death (SCD), whole-exome sequencing

## Abstract

Cellular action potential is characterized by a particular sequence of depolarizing and repolarizing ion currents regulated by ion channels. Genetic mutations in these channels disrupt the essential movement of ions, such as Na+, Ca++, and K+, across the cell membrane, leading to dangerous arrhythmias and sudden cardiac death (SCD). Most cases of unexplained SCD are caused by pathogenic variants in genes linked to channelopathies and cardiomyopathy. Genetic investigations might aid in confirming the clinical diagnosis based solely on observations. Other advantages of genetic studies are clinical management of the patient, family screening, appropriate genetic counseling, and risk assessment for family members. This study was conducted to investigate the genetic cause of early‐onset SCD in two Iranian families. Whole‐exome sequencing was performed on the probands from each family, and the Illumina DRAGEN haplotype variant calling system was used to identify variants in each patient. Here, we identified rare heterozygous missense variants in the *RYR2* and *SCN5A* genes, which are linked to cardiac channelopathies. Alignment studies reveal that the mutated residues are conserved across humans and primates, underscoring their crucial role in protein function. Previously reported associations between these mutations and channelopathy pathogenesis have been confirmed in the present study. This study provides valuable insights for genetic counseling of families with a history of sudden death.

## 1. Introduction

Ion channels play a crucial role in the heart′s proper function. Abnormal function of these channels, known as cardiac ion channelopathies, can lead to sudden cardiac death (SCD). SCDs may have a genetic origin, and roughly 40%–50% of rare genetic variants associated with SCDs impact electrical membrane ion channels, which are essential for maintaining heart electrical activity and managing intracellular calcium (Ca2+) balance. The cellular action potential of the heart during its cycle is determined by depolarizing and repolarizing ion currents, which are regulated by ion channels involving calcium (Ca2+), sodium (Na+), and potassium (K+) ions [1]. Furthermore, an increasing number of mutations in the *SCN5A* gene, which encodes the pore‐forming *α*‐subunit of the primary cardiac sodium channel (NaV1.5), have been found in patients with ECG abnormalities and various cardiac syndromes [2]. According to the disease‐related genetic variations database “ClinVar” (ClinVar, 2024), approximately 517 likely pathogenic and pathogenic variants in the *SCN5A* gene are associated with various cardiovascular disorders. Mutations in *SCN5A* cause heart abnormalities, including Brugada syndrome, long QT syndrome (LQTS) Type 3, sick sinus syndrome, atrial flutter, atrial fibrillation, dilated cardiomyopathy (DCM), and other similar phenotypes and syndromes. Indeed, the same ion channel mutations can manifest in various phenotypes [3]. The ryanodine receptor (*RyR*), another gene associated with channelopathies, is the largest calcium release channel within cells, located on the sarcoplasmic reticulum/endoplasmic reticulum (SR/ER), and plays a pivotal role in regulating calcium (Ca2+) levels in cells. Similar to *SCN5A*, mutations in ryanodine receptors (*RyR2*) have been reported in cardiac diseases [4]. Mutations related to *RyR2* are categorized as gain‐of‐function (GOF) or loss‐of‐function (LOF), each corresponding to distinct clinical manifestations. GOF alterations in the *RYR2* gene are known as a cause for catecholaminergic polymorphic ventricular tachycardia (CPVT) and cause increased spontaneous calcium release, contributing to ventricular arrhythmias and SCD. The mutations that cause LOF of the *RYR2* channel are linked to a separate form of cardiac arrhythmia called RyR2 Ca2+ release deficiency syndrome (CRDS), which differs from CPVT caused by GOF *RYR2* mutations [5, 6]. The Heart Rhythm Society and European Heart Rhythm Association guidelines recommend comprehensive or targeted genetic testing of ion channels (specifically, *KCNQ1*, *KCNH2*, *RYR2*, and *SCN5A)* to identify the cause and type of SCDs that exhibit no postmortem signs. Detection of genetic causes not only aids in preventing death but also facilitates the labeling of relatives who are at risk of heart disease and allows for valuable genetic counseling [7]. At the Genetics Research Center, University of Social Welfare and Rehabilitation (USWR), we focused on identifying underlying genetic causes of familial SCD and cardiac disease. Discovering genes and variants that cause SCD enables the performance of targeted sequencing (a multigene NGS panel) or targeted analysis, resulting in reduced costs, shorter diagnosis times, and increased accuracy and confidence in the diagnosis. However, the genetic factors that contribute to familial SCD are unknown. Mutations in well‐known genes account for only a small percentage of familial SCD, whereas the majority of mutations in other genes remain uncovered [8]. In the current study, we aimed to find the undiscovered genetic causes of familial SCD in two large Iranian pedigrees.

## 2. Materials and Methods

### 2.1. Case Presentation

In Family 1, the proband was a 62‐year‐old female with angiographically proven coronary artery disease (CAD), three affected siblings, and two unaffected ones (Figure 1a). SCD of unknown cause was reported in her mother and several second‐degree relatives from her maternal lineage. The proband had experienced two prior episodes of cardiac attacks at 23 and 49 years of age, with an abnormal ECG during her cardiac attack (presumably suggestive of arrhythmia). Clinical assessments showed no signs of CAD in affected family members, as angiography results were negative, yet they experienced early‐onset myocardial infarction.

Figure 1(a) Pedigree of the family, (b) cosegregation results of *RYR2* variant (NM_001035.3) (c.9778C > T) in Family 1 using Sanger sequencing, and (c) *RYR2* protein sequence alignment. The number 25 indicates the amino acid count in the sequence. The arrow indicates arginine, which is highly conserved among close species.

**Figure:**
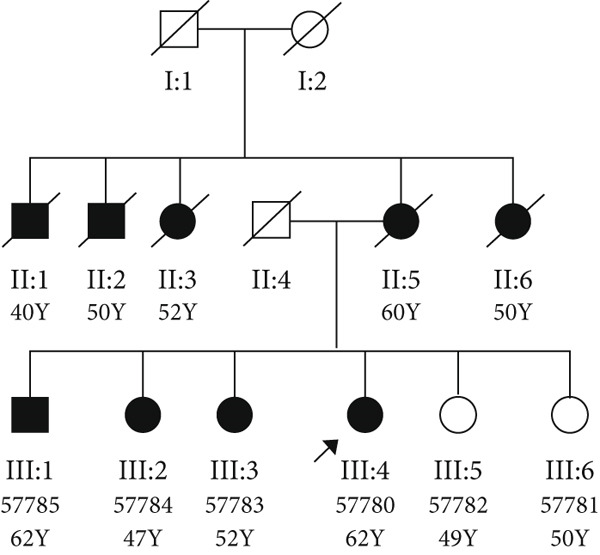
(a)

**Figure:**
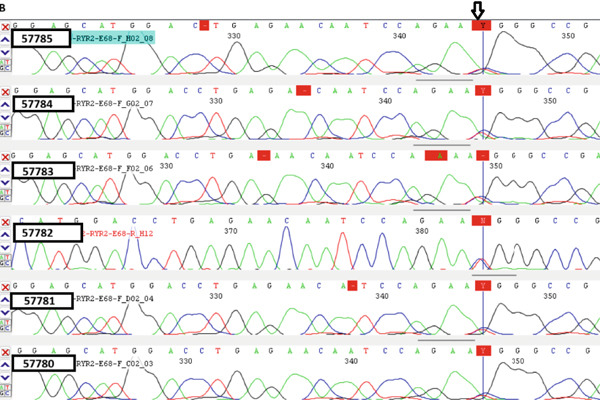
(b)

**Figure:**
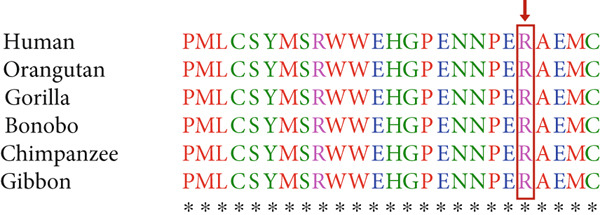
(c)

The proband of Family 2 was a 38‐year‐old woman suspected of LQTS with a history of previous SCD in her family. Two of her siblings passed away at the ages of 48 and 37. The patient stated that the mother also died from cardiac complications at the age of 40. The pedigree manifests an autosomal dominant pattern of inheritance (Figure 2a).

Figure 2(a) Pedigree of the family, (b) Sanger sequencing of the *SCN5A g*ene revealed (NM_000335.5) c.5347G > A variant in the family, and (c) *SCN5A* protein sequence alignment. The number 25 indicates the amino acid count in the sequence. The arrow indicates arginine, which is highly conserved among close species.

**Figure:**
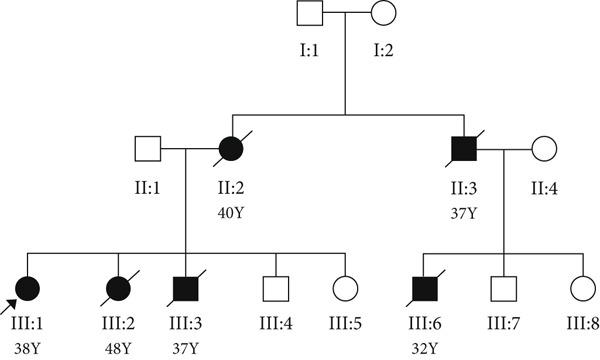
(a)

**Figure:**
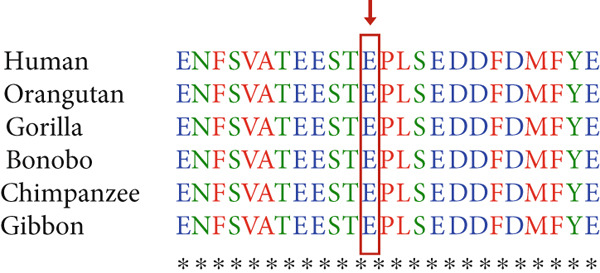
(b)

### 2.2. Whole‐Exome Sequencing and Variant Confirmation

Peripheral blood samples were collected from the probands, their parents, and the other affected and healthy siblings. All participants provided informed consent prior to taking part in the study. The project received approval from the Ethics Committee for Human Research at the University of Social Welfare and Rehabilitation Sciences (IR.USWR.REC.1402.188) and was carried out in accordance with applicable ethical standards. DNA samples were extracted from the peripheral blood using the salting‐out method, and DNA quality was examined by a NanoDrop Spectrometer (Thermo Scientific, United States).

For each proband, paired‐end sequencing (Illumina NextSeq 500 platform, Illumina, San Diego, California, United States) was done after exome capturing by the Agilent SureSelectXT2 Kit (Version 6) (Agilent Technologies, Inc., Santa Clara, California). Quality control analysis was conducted using the FastQC toolkit, and the data were aligned to the human reference genome build GRCh37 (hg19) utilizing the Illumina DRAGEN Bio‐IT Platform. Identification of single‐nucleotide variants, small deletions, and insertions was performed by the Illumina DRAGEN haplotype variant calling system. The found variants were then annotated using VarSeq v2 software (Golden Helix, Inc., Bozeman, Montana, http://www.goldenhelix.com). The identified variants were initially filtered based on variant quality (> 30) and depth (> 5). Then, according to allele frequencies reported in population databases such as the Genome Aggregation Database (gnomAD) (https://gnomad.broadinstitute.org), the 1000 Genome Project (http://www.1000genomes.org), and Iranome (http://www.iranome.ir), variants with a minor allele frequency of over 0.01 were excluded. Furthermore, the remaining variants were prioritized according to variant type (missense, LOF, and the other ones (synonymous and noncoding variants), and scores obtained from in silico prediction tools including SIFT, CADD (Combined Annotation Dependent Depletion), Polyphen‐2, and so on. Ultimately, variants were classified based on the American College of Medical Genetics and Genomics (ACMG) guidelines. The protein sequences of *RYR2* and *SCN5A* in humans and five nonhuman primate species (orangutan, gorilla, bonobo, chimpanzee, and gibbon) were retrieved from Ensembl genome browser release 113 (http://ensembl.org). These species were selected as representatives of the Hominidae family (great apes), which are the closest evolutionary relatives to humans. Protein sequence conservation among these species was analyzed using Clustal Omega to generate multiple sequence alignments and assess evolutionary conservation within this primate group.

### 2.3. Sanger Sequencing

The confirmation of the candidate variant and the cosegregation analysis were conducted using Sanger sequencing. PCR primers for the candidate mutation were designed using the reference sequence from UCSC and were synthesized by the Sina Colon Company. The PCR conditions were as follows: 94°C for 3 min, 94°C for 30 s, 55°C for 30 s, and 72°C for 60 s for 30 cycles, and 72°C for 5 min. Sanger sequencing was carried out using the ABI 3130 Sequencer (Life Technologies, Carlsbad, California, United States).

## 3. Results

We identified two rare heterozygous missense variants, previously reported, in the *RYR2* and *SCN5A* genes in two unrelated families. Confirmation of candidate variants identified with WES was carried out by standard PCR and Sanger sequencing. The first missense variant, c.9778C > T (p. Arg3260Trp) in the *RYR2* gene, was detected in the proband of Family 1 in heterozygous form (Table 1). Cosegregation analysis in Family 1 showed that other affected members of the family also have the same genotype of c.9778C > T (heterozygote), which was found in the proband. The seemingly healthy siblings also harbor the mutation (Figure 1b). This variant was classified as of uncertain significance (VUS) according to ACMG guidelines, based on its rarity in population databases such as gnomAD (PM2), and its location within the *RYR2* gene, which has a low rate of benign missense variation (PP2) [9].

**Table 1:** Detailed information for candidate variants.

**Gene**		***RYR2* (NM_001035.3)**	***SCN5A* (NM_000335.5)**
Variant		Chr1:237870446C > T c.9778C > T p.Arg3260Trp	Chr3:38592513 C > T c.5347G > A p.Glu1783Lys
dbSNP		rs765325906	rs137854601
Main clinical feature and age of onset		Arrhythmia and early onset of coronary artery disease (23 y/o)	Arrhythmia (38 y/o)
ACMG classification		Uncertain significance	Pathogenic
Allele frequency	1000 genome	Not reported	Not reported
	GnomAD	0.000055 Very rare	0.000008 Very rare
	Iranome	Not reported	Not reported
	ExAC	0.0042 Very rare	Not reported
In silico prediction tools	SIFT	Uncertain	Uncertain
	Polyphen	Uncertain	Deleterious
	fitCons	Deleterious	Deleterious
	DANN	Deleterious	Deleterious
	CADD phred score	18	26

Also, we identified a heterozygous pathogenic missense variant, c.5347G > A (p. Glu1784Lys), in the *SCN5A* gene in the index patient of the second family. The identified variant in the *SCN5A* gene was classified according to ACMG guidelines using multiple criteria. Functional studies demonstrated a damaging effect on the NaV1.5 sodium channel, which *SCN5A* encodes. The variant is located within a known mutational hotspot and critical functional domain. It is extremely rare in population databases such as gnomAD. Computational predictions consistently indicate a harmful impact, and a reputable source has recently classified the variant as pathogenic. Together, these lines of evidence support classifying the variant as pathogenic (PS3, PM1, PM2, PP3, PP5) [9]. Still, the family members did not cooperate in further studies to confirm the cosegregation analysis.

The alignment study of the residues among humans and primates shows that residues are conserved positions in protein sequences (Figures 1c, 2b).

## 4. Discussion

SCD is generally caused by a fatal ventricular arrhythmia that progresses to death within a few minutes [10]. Ischemic heart disease is often the leading cause of this condition. However, for those under 35 years old, the primary causes are usually cardiomyopathy and channelopathies, both of which have a genetic basis. The complexity of this genetic cause is increasing over time, characterized by significant diversity and an expanding number of responsible genes [11–13]. For familial cases, genetic testing allows for risk assessment in first‐degree relatives. Therefore, genetic counseling is highly advised for family members with cases of a heart disease‐causing mutation, enabling timely interventions and disease management. Identifying specific genes and variants associated with the disease allows for the development of targeted genetic panels, causing earlier and more cost‐effective diagnosis of heart‐related disorders. This approach not only aids in detecting population‐specific mutations and providing more effective genetic counseling but also enhances understanding of the disease′s underlying mechanisms. Consequently, this can improve disease management and assist in selecting the most appropriate treatment options for affected patients. So, the findings from this study may facilitate personalized therapies for familial SCD cases and help design new drug targets in the future [14–16].

This study is aimed at identifying the cause of familial SCD in two large Iranian families with a familial history of sudden death with no apparent causative factor. Next‐generation sequencing (NGS) identified two previously reported heterozygous rare variants, c.9778C > T and c.5347G > A, in the *RYR2* and *SCN5A* genes, respectively.

The cardiac *RyR2*, encoded by the *RYR2* gene that contains 105 exons, is one of the most prominent ion channel proteins, comprising 4967 amino acids, and is located at the SR, playing a crucial role in regulating the release of calcium within cells and facilitating cardiac muscle contraction. CPVT is the primary condition related to *RYR2* mutations and is considered to be stress‐caused ventricular arrhythmias and SCD in hearts with normal structure. The latest high‐resolution cryo‐electron microscopy (EM) researches disclose that the long *RyR* polypeptide chain is arranged into separate structural regions, primarily composed of *α*‐helices and oriented toward the cytoplasm. These regions contain the N‐terminal domain (NTD), which consists of Subdomains A, B, and C; three SPRY domains, including the bridging solenoid (BSol), the junctional solenoid (JSol), *RyR* repeats 1 and 2 (RY1 and 2) with Subdomains B and C flanking RY3 and RY4; and the core solenoid (CSol). The C‐terminal contains about 500 amino acids and consists of the transmembrane domain (TMD) containing six segments that maintain the protein on the SR membrane, with a short cytosolic CTD. Although *RYR2* alterations are spread throughout the gene, the majority of them are concentrated in four hot spots: residues 44–466 are located in the NTD, residues 2246–2534 in the helical domain, 3778–4201 in the central domain, and 4497–4959 in the channel domain. The p.R3260W variant (c.9778C > T), found in coding Exon 68 of the *RYR2* gene, is between 2976 and 3527 residues. This domain encompasses 19.8% of the mutations [12].

The identified substitution replaces arginine at codon 3260 with tryptophan, an amino acid with differing characteristics. Replacing a positively charged arginine with a large and hydrophobic amino acid, tryptophan, may disrupt the protein′s structure or behavior, potentially impairing channel gating and stability. Such alterations can cause deregulated calcium release during cardiac muscle contractions, subsequently contributing to arrhythmias and other heart‐related diseases. Comparison of the protein sequence of *RYR2* in humans and other species showed that the found mutation is located in the conserved region of the protein, supporting the idea that this missense mutation may be a deleterious variant due to alteration of the important regions of proteins.

Computational prediction, such as SIFT and Polyphen, suggests that this variant has a VUS effect on protein function; however, fitCons, DANN, and M‐CAP predict a deleterious impact on protein function. To our knowledge, there have been no reported functional studies concerning this variant. However, our study supports the destructive impact of the mutation c.9778C > T in *RYR2* in heart‐related disease. Then again, we did not find any other heart disease‐related mutation in the patient, which increases the possibility that c.9778C > T is the causal variant we were going to find.

Various symptoms have been reported in cases with a mutation in *RYR2*, one of which is CPVT, marked by episodes of potentially life‐threatening bidirectional or CPVT induced by stress, manifesting without structural heart abnormalities and normal ECG findings at rest [5]. The *RYR2* gene mutations are also a cause of arrhythmogenic right ventricular cardiomyopathy (ARVC2) with clinical manifestations similar to CPVT, such as stress‐induced ventricular tachycardia, in addition to ongoing deterioration and fibro–fatty infiltration of the right ventricle. Moreover, *RYR2* alterations are known to be linked to nonstress‐induced cardiac diseases such as left ventricular noncompaction, LQTS, DCM, torsade de pointes, and hypertrophic cardiomyopathy (HCM) [17].

The variant has been previously reported in ClinVar and the literature as a VUS with different phenotypes. This variant was reported in 2017 as a candidate novel variant in a study that employed whole‐exome sequencing to identify hereditary cardiovascular diseases, particularly in poorly defined cases of SCD. It was found in an individual with a cardiovascular phenotype, cardiomyopathy, atrial arrhythmia, and neuromuscular disorder [18]. Additionally, this variant was found in another cohort study examining genes linked to catecholaminergic CPVT, based on a large group of patients referred for clinical whole‐exome genetic testing [19].

In our patient, the mutation in *RYR2* manifests itself through a distinct set of symptoms, namely, a characteristic arrhythmic phenotype, an abnormal ECG, and a cardiac attack and CAD. This observation represents the phenomenon of phenotypic heterogeneity associated with the *RYR2* gene. A previous study suggested that the *RyR2* gene may be a candidate gene involved in familial early‐onset CAD. [20]. Dysfunction in the *RyR2* gene and intracellular calcium signaling contribute to inflammation through immune cells, such as T lymphocytes, and pro‐inflammatory signaling pathways [21]. Inflammation is a central contributor to the pathogenesis of CAD, where immune cells infiltrate vascular tissue, secrete inflammatory cytokines, and accelerate the formation and instability of atherosclerotic plaques [22]. This could explain the CAD phenotype in patients in this family. Cosegregation analysis showed the presence of a heterozygous mutation c.9778C > T in patients with the cardiac disease. A follow‐up CCTA examination revealed that a healthy sibling (#57782, III: 5) carrying the mutation had coronary stenosis, indicating that another seemingly healthy sibling with the carrier genotype in the family may also be at risk of developing the disease in the future (see genotypes in Figure 1).

In another family, we identified a known pathogenic variant c.5347G > A (p.Glu1784Lys) in the *SCN5A* gene. This variant, 17 out of 26 submissions, contributed to pathogenic classification based on the ACMG guideline. A comparison study represented that this mutation is located in a conserved position of the *SCN5A*, supporting its adverse effects on the protein properties and activity. Heart arrhythmias, such as LQTS Type 3, Brugada syndrome, sick sinus syndrome, atrial fibrillation, atrial flutter, DCM, and more phenotypically heterogeneous and overlapping syndromes, are developed by alterations in *SCN5A* [23]. These conditions arise because the *SCN5A* mutations cause abnormalities in how the cardiac sodium channel NaV1.5 opens and closes. Specifically, mutations can either increase or decrease the inward sodium current (I_Na). GOF mutations impair the channel′s fast inactivation, leading to an increased persistent (late) sodium current that prolongs the cardiac action potential, which is characteristic of LQT3. On the other hand, LOF mutations reduce the peak sodium current, which mainly causes Brugada syndrome by impairing electrical conduction. Notably, specific mutations can produce both GOF and LOF effects simultaneously, contributing to overlapping clinical syndromes. A substitution from guanine to adenine at position 5349 in the *SCN5A* gene results in the E1784K mutation, which changes the charge of an amino acid in the C‐terminal domain (CTD) of the NaV1.5 channel. This mutation is the most frequently found variant associated with mixed syndromes. Clinical studies show that individuals carrying E1784K can exhibit a wide range of symptoms. The E1784 residue is located just before the acidic EF‐like hand domain (helices *α*1–*α*4). It plays a role in the electrostatic interaction between this acidic region and the nearby basic IQ domain (*α*_6_). The CTD where E1784K occurs has a strong influence on how the channel opens and inactivates. The charge change caused by E1784K is believed to disrupt the stability of the CTD, making the *α*6 helix more flexible. This increased mobility of *α*6 is linked to higher late sodium current (late I_Na) and more substantial slow inactivation—critical changes that underlie the mutation′s effects on channel function [24]. The identified variant, p.Glu1784Lys, was associated with a sustained inward sodium current and an accelerated recovery from inactivation, indicating a disruption of the inactivation state. This continuous current may slow down the repolarization process of the action potential and enhance the risk of torsade de pointes, potentially causing syncope or sudden death. This variant was initially reported in patients presenting with mild bradycardia, LQTS, and SCD by Wei et al. and subsequently by several other groups [25–27]. Tester et al. indicated that c.5347G > A is the most prevalent mutation in genetic screening of 541 patients with LQTS [28]. Takahashi et al. performed targeted sequencing of *KCNQ1*, *KCNH2*, and *SCN5A* in 23 students suspected of having LQTS as part of a school‐based ECG screening program. Interestingly, 17 patients were found to have a genetic mutation; among them, 14 were identified as carriers of the *SCN5A* E1784K [29]. Makita et al. genetically analyzed 44 LQT3 families with multiple ethnicities and reported the E1784K mutation in *SCN5A* as a commonly identified mutation that is responsible for up to 34% of LQT3 cases [30]. Our results in the present study confirm the previously reported associations of the two *RYR2* and *SCN5A* variants in the pathogenesis of SCD. However, additional research with larger pedigrees and functional studies is necessary to establish the pathogenicity and clinical relevance of variants.

## 5. Conclusion

This study reports the discovery of two rare variants in the *SCN5A* and *RYR2* genes in two unrelated Iranian families. Identifying these variants enables effective genetic counseling for the families. The study suggests mutation analysis of channelopathy–related genes in patients with familial SCD and seemingly healthy young individuals of the family. This will help in the early diagnosis and management of the disease. Although our study successfully identified rare heterozygous variants in *RYR2* (c.9778C > T) and *SCN5A* (c.5347G > A) genes associated with familial SCD, the lack of direct functional validation for the *RYR2* gene limits definitive conclusions about its pathogenicity. Future studies using functional assays to examine calcium release dynamics could clarify how *RYR2* variants affect calcium handling in cardiomyocytes. Complementary in silico analyses, along with molecular modeling, may also enhance pathogenicity evaluations and guide experimental prioritization. These approaches will be essential to validate the clinical significance of the identified variant, which may be the focus of our future studies.

## Disclosure

All authors reviewed and accepted the final manuscript.

## Conflicts of Interest

The authors declare no conflict of interest.

## Author Contributions

Mahsa Tahmasebivand: manuscript drafting and exome sequencing data analysis; Sepideh Mehvari: family recruitment, providing samples and the informed consent forms, exome sequencing data analysis, and manuscript revision; Fatemeh Ghodratpour: primer design, Sanger sequencing, and cosegregation study; Reza Najafipour: scientific consultation and financial support. Reza Malekzadeh, Yasser Riazalhosseini, Mark Lathrop, Hamidreza Khoram Khorshid, and Hossein Najmabadi: study design, scientific consultation, and critical manuscript revision; and Kimia Kahrizi: study design, manuscript revision, genotype–phenotype correlation, obtained funding, and principal investigator.

## Funding

This work was funded by the Genetics Research Center (801A/6/45329) and the Iran National Science Foundation (INSF) (10.13039/501100003968) (96011200).

## Data Availability

The data supporting the conclusions of this study are accessible from the corresponding authors upon reasonable request.
